# Supplementary material for: Relationships between body fat distribution and metabolic syndrome traits and outcomes: A mendelian randomization study
Source: PLoS One. 2023 Oct 26;18(10):e0293017. doi: 10.1371/journal.pone.0293017 (PMC10602264; doi:10.1371/journal.pone.0293017)
Supplement: S1 Table — Details regarding the GWAS studies referenced for each cardiometabolic outcome trait are displayed. Abbreviations: GLGC (Global Lipids Genetics Consortium), UKB (United Kingdom BioBank), MAGIC (Meta-Analyses of Glucose and Insulin-related traits Consortium), DIAGRAM (Diabetes Genetics Replication And Meta-analysis), GERA (Genetic Epidemiology Research on Adult Health and Aging), ICBP (International Consortium for Blood Pressure). (DOCX) [file pone.0293017.s001.docx]

| Trait | Group | Year | PMID | Sample Size |
| --- | --- | --- | --- | --- |
| LDL cholesterol | GLGC | 2013 | 24097068 | 173,082 |
| Apolipoprotein B | UKB | 2020 | 32202549 | 439,214 |
| HDL cholesterol | GLGC | 2013 | 24097068 | 187,167 |
| Apolipoprotein A-1 | UKB | 2020 | 32202549 | 393,193 |
| Total Cholesterol | GLGC | 2013 | 24097068 | 187,365 |
| Triglycerides | GLGC | 2013 | 24097068 | 177,861 |
| Fasting Glucose | 130 cohorts | 2021 | 34059833 | 200,622 |
| HbA1C | MAGIC | 2010 | 20858683 | 46,368 |
| Type 2 Diabetes | DIAGRAM, GERA, and UKB | 2018 | 30054458 | 655,666 |
| Systolic blood pressure | ICBP | 2018 | 30224653 | 757,601 |
| Diastolic blood pressure | ICBP | 2018 | 30224653 | 757,601 |
