# Supplementary material for: Relationships between body fat distribution and metabolic syndrome traits and outcomes: A mendelian randomization study
Source: PLoS One. 2023 Oct 26;18(10):e0293017. doi: 10.1371/journal.pone.0293017 (PMC10602264; doi:10.1371/journal.pone.0293017)

**a) Univariate Type 2 Diabetes MR (Absolute fat depots and relative ratios)**


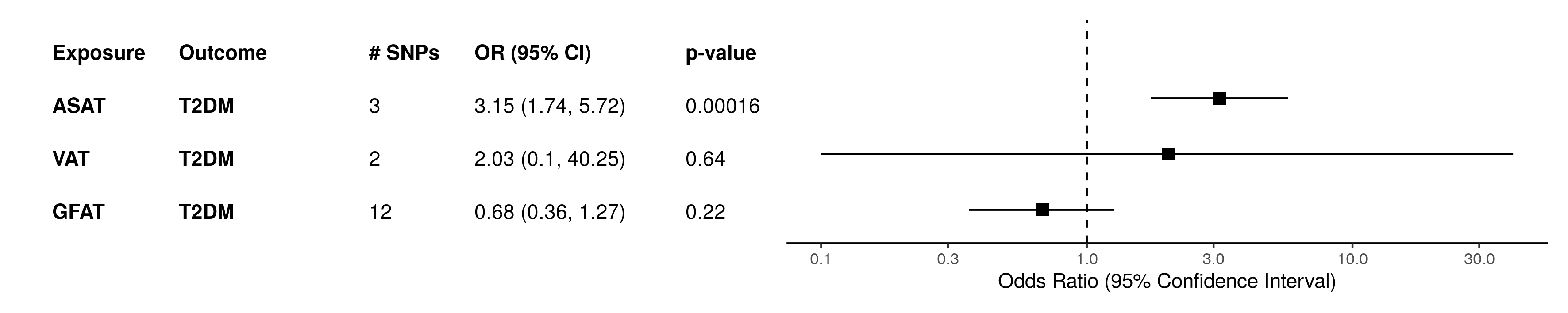

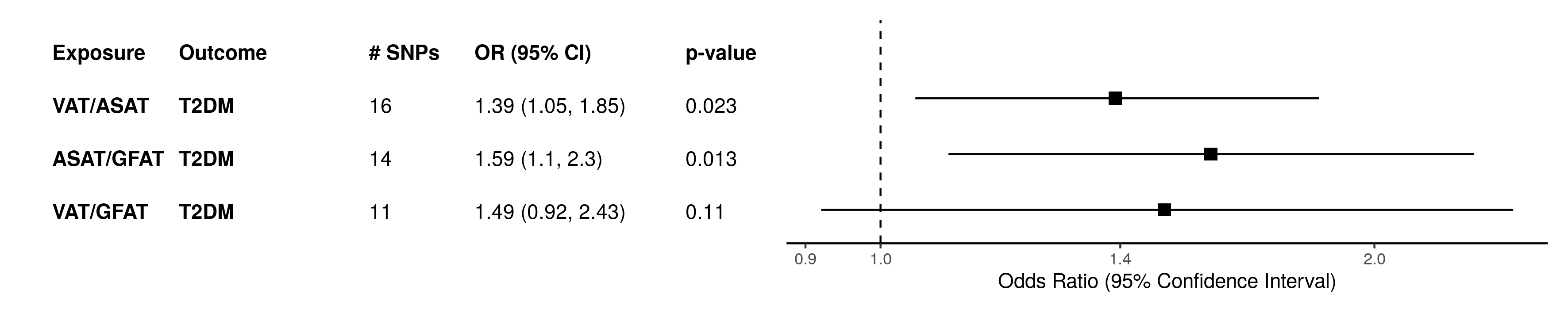


**b) Type 2 Diabetes MVMR (Controlled for BMI)**


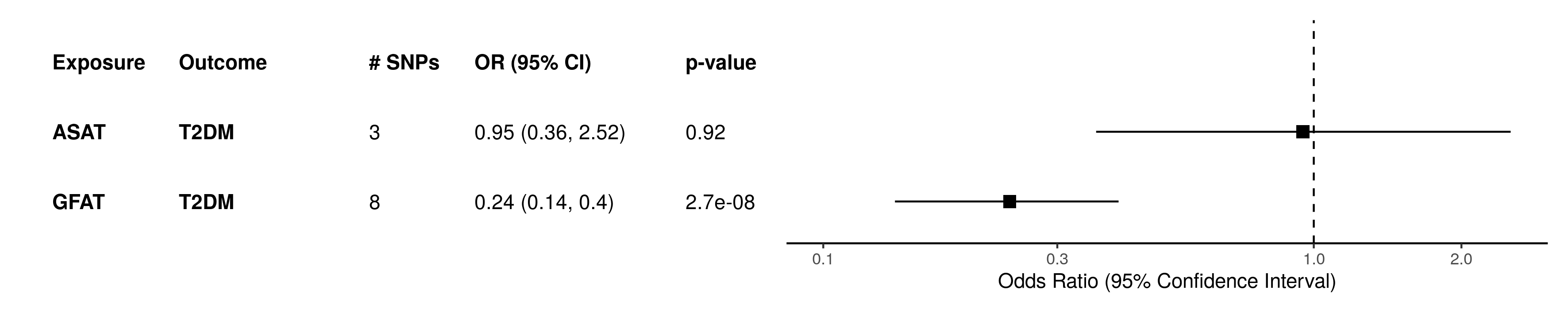

Supplement: S1 Fig — Results for univariate mendelian randomization (MR) with absolute fat depot values and their relative ratios as exposures and type 2 diabetes (T2DM) as the outcome are shown in a). Results for multivariable mendelian randomization (MVMR) for the absolute fat depots controlling for BMI are shown in b). MR and MVMR estimates were calculated with inverse variance weighting and logarithmic scaling was applied to the x-axis to display confidence intervals. # SNPs denotes the number of SNPs used as instruments from each exposure GWAS. Abbreviations: ASAT (abdominal subcutaneous adipose tissue), GFAT (gluteofemoral adipose tissue), VAT (visceral adipose tissue). (DOCX) [file pone.0293017.s003.docx]
