# Supplementary figures and images for: Relationships between body fat distribution and metabolic syndrome traits and outcomes: A mendelian randomization study
Source: PLoS One. 2023 Oct 26;18(10):e0293017. doi: 10.1371/journal.pone.0293017 (PMC10602264; doi:10.1371/journal.pone.0293017)

**
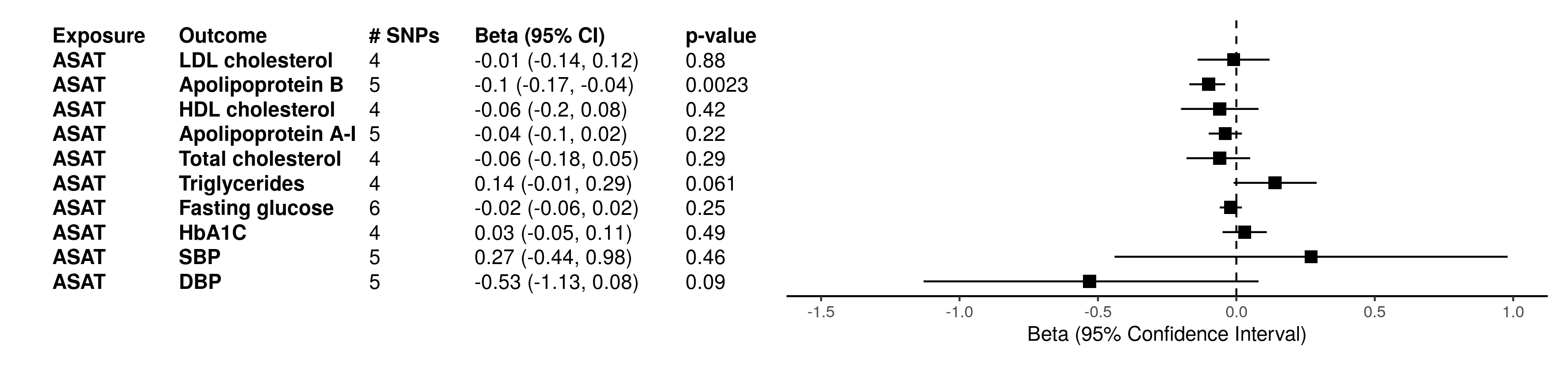

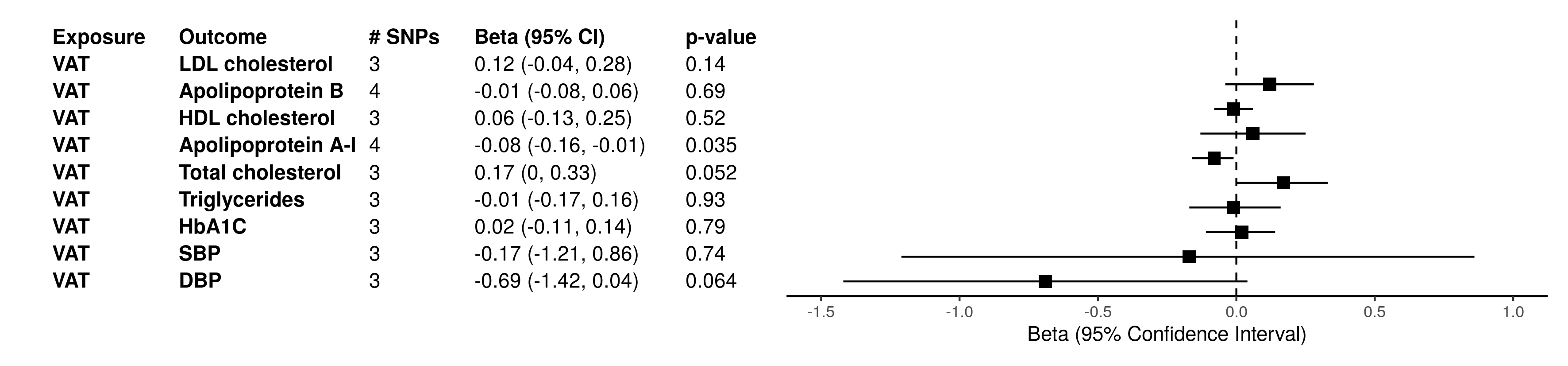

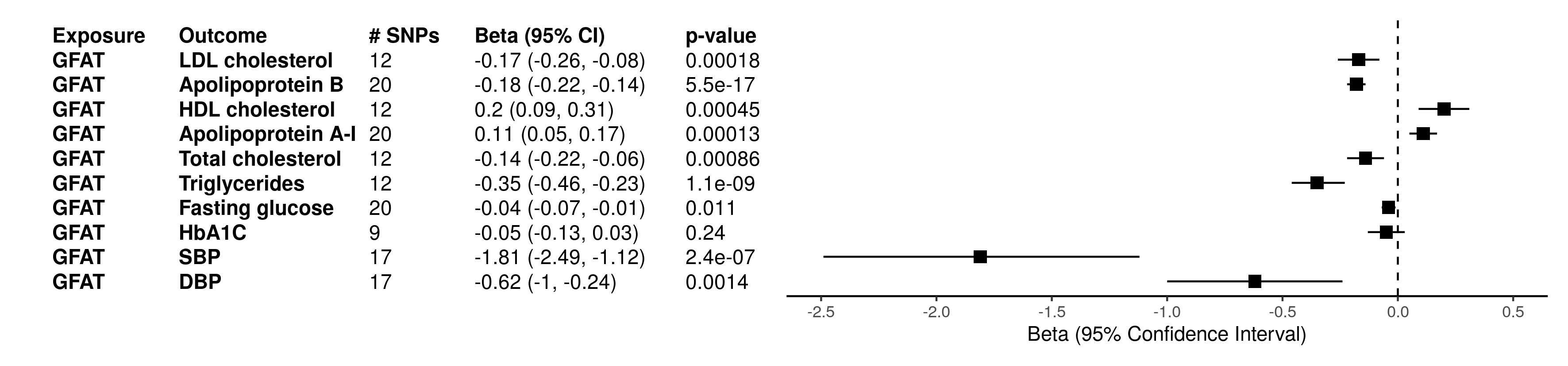

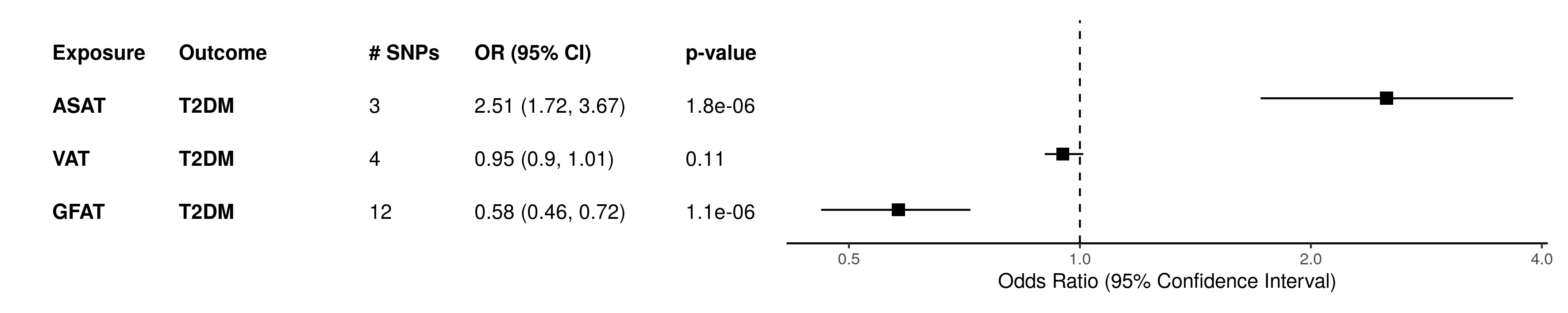
**

Supplement: S2 Fig — Results for univariate mendelian randomization (MR) with absolute fat depots as exposures and cardiometabolic markers as outcomes using weighted median estimates. Logarithmic scaling was applied to the x-axis for the Type 2 diabetes outcome MR. Abbreviations: ASAT (abdominal subcutaneous adipose tissue), GFAT (gluteofemoral adipose tissue), VAT (visceral adipose tissue). SBP (systolic blood pressure), DBP (diastolic blood pressure. (DOCX) [file pone.0293017.s004.docx]

**
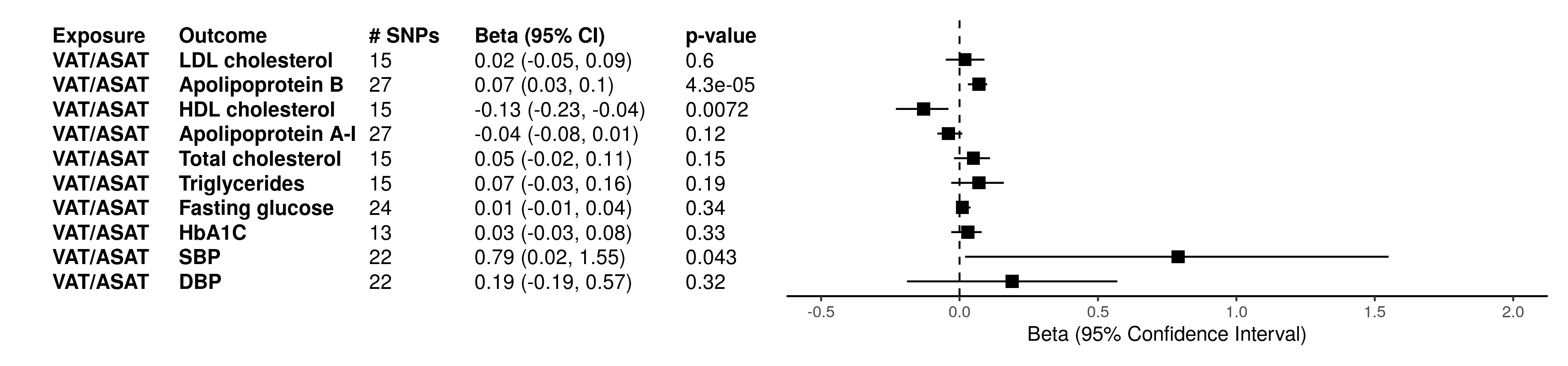

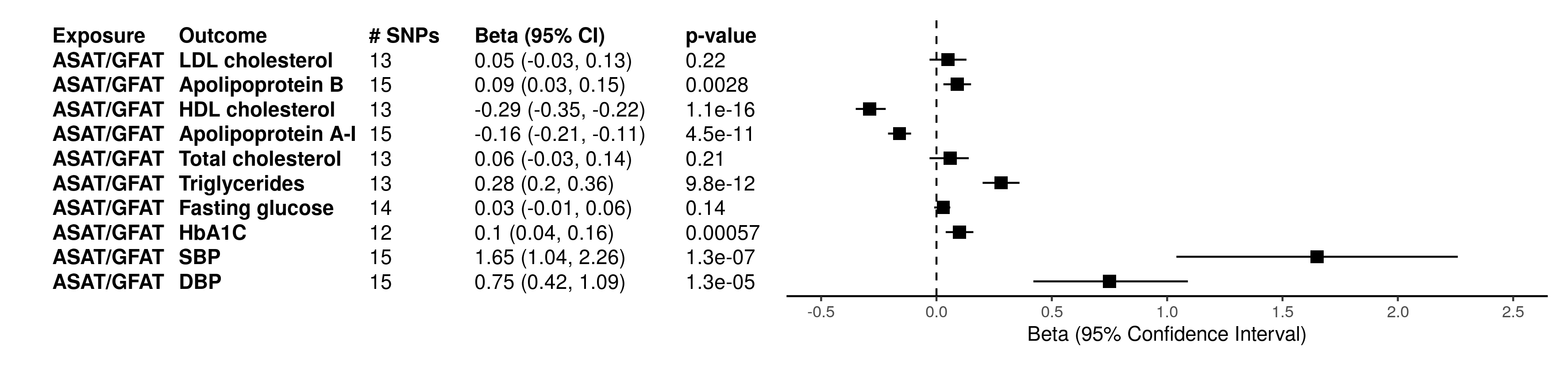

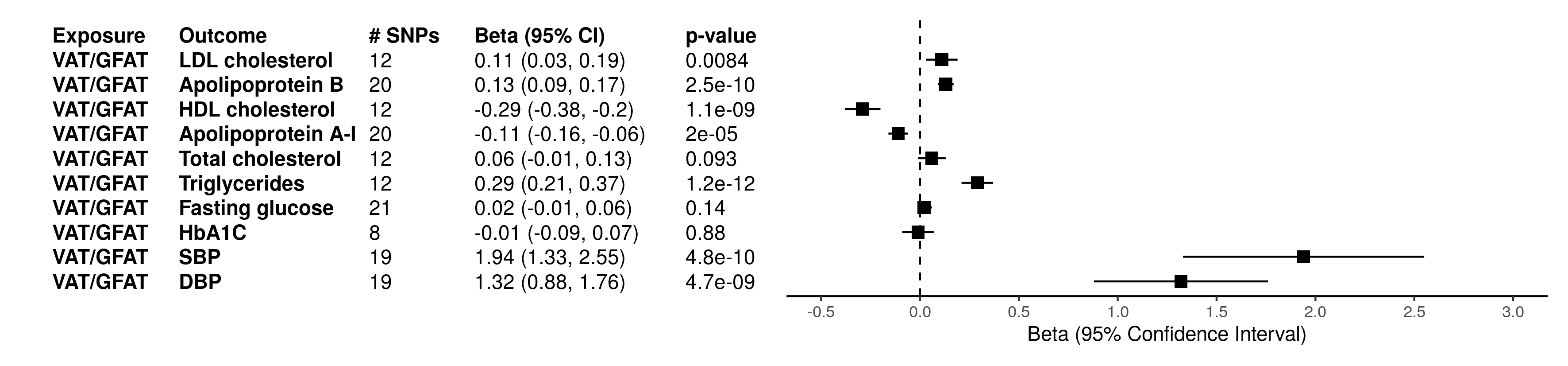

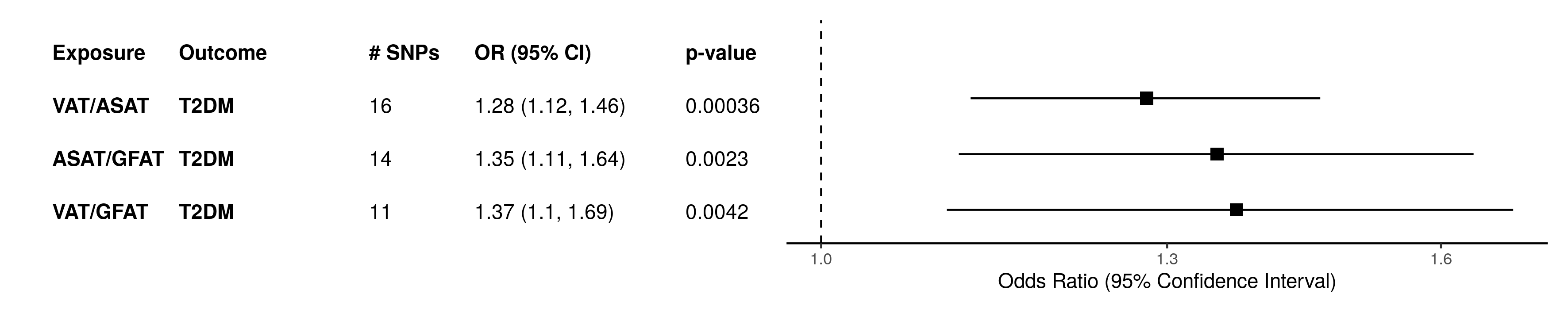
**

Supplement: S3 Fig — Results for univariate mendelian randomization (MR) with relative fat ratios as exposures and cardiometabolic markers as outcomes using weighted median estimates. Logarithmic scaling was applied to the x-axis for the Type 2 diabetes outcome MR. Abbreviations: ASAT (abdominal subcutaneous adipose tissue), GFAT (gluteofemoral adipose tissue), VAT (visceral adipose tissue). SBP (systolic blood pressure), DBP (diastolic blood pressure). (DOCX) [file pone.0293017.s005.docx]
