# Supplementary material for: Relationships between body fat distribution and metabolic syndrome traits and outcomes: A mendelian randomization study
Source: PLoS One. 2023 Oct 26;18(10):e0293017. doi: 10.1371/journal.pone.0293017 (PMC10602264; doi:10.1371/journal.pone.0293017)
Supplement: S4 Fig — Inverse variance weighted mendelian randomization estimates excluding the indicated SNP were calculated for each SNP and pair of exposures and outcomes. VAT on T2DM leave-one-out analyses was unable to be performed due to insufficient number of genetic instruments for VAT. Estimates obtained with all SNPs included are also displayed at the bottom of each graph. ASAT, VAT, GFAT, VAT/ASAT, ASAT/GFAT, and VAT/GFAT are shown in parts a), b), c), d), e), and f) respectively. Abbreviations: ASAT (abdominal subcutaneous adipose tissue), GFAT (gluteofemoral adipose tissue), VAT (visceral adipose tissue). SBP (systolic blood pressure), DBP (diastolic blood pressure. (DOCX) [file pone.0293017.s006.docx]

a) ASAT leave-one-out analyses.
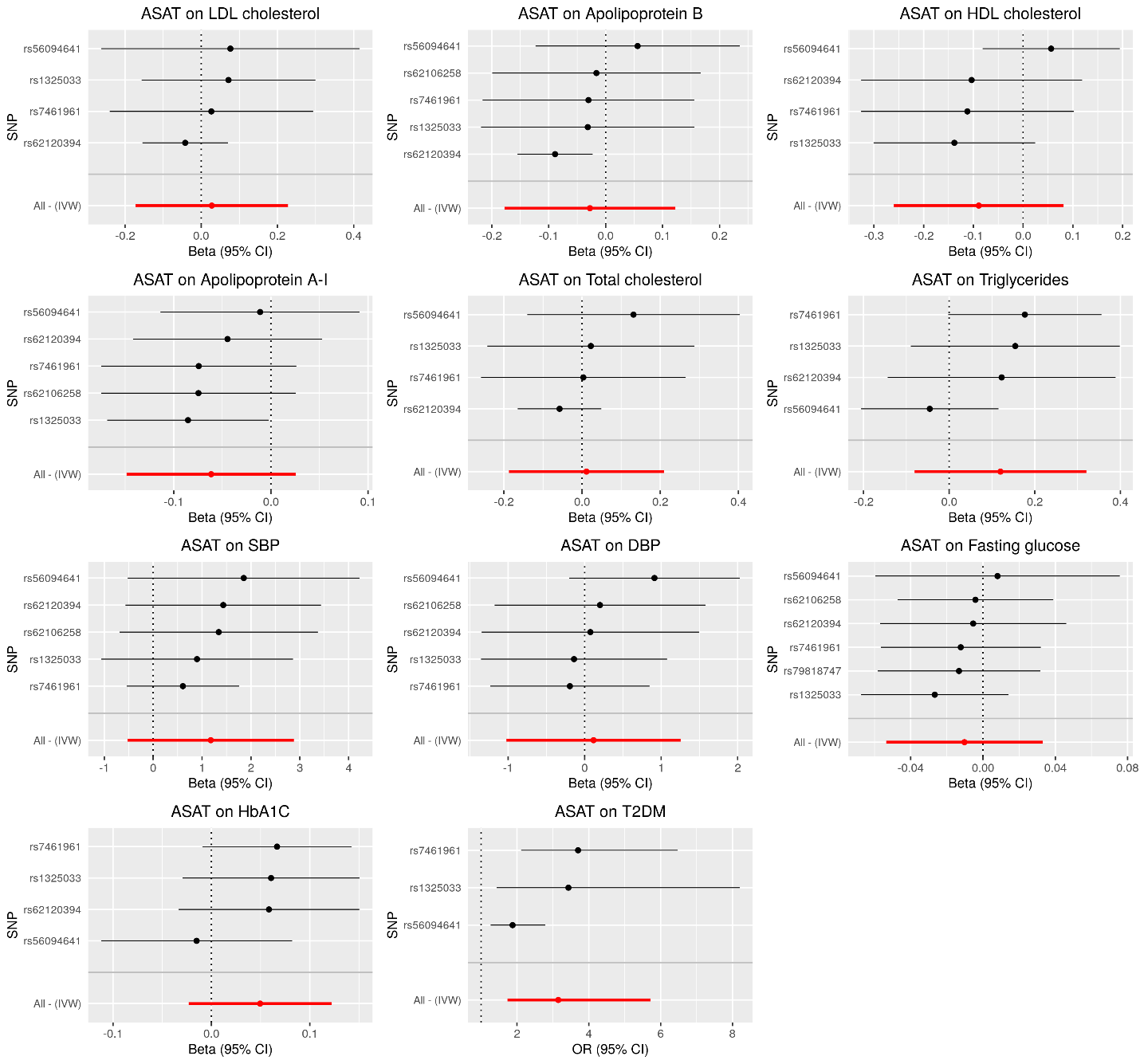


b) VAT leave-one-out analyses.
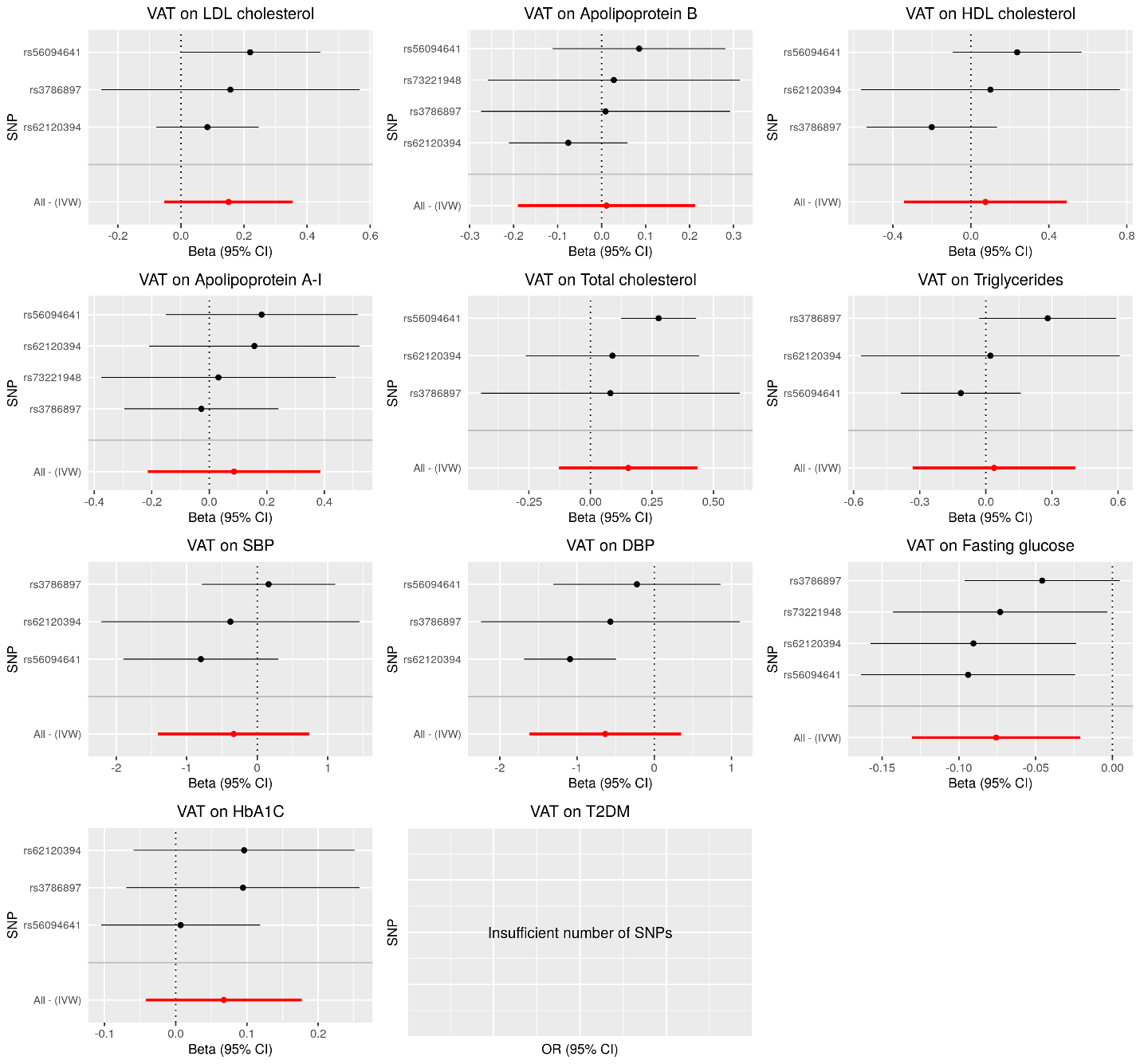


c) GFAT leave-one-out analyses.
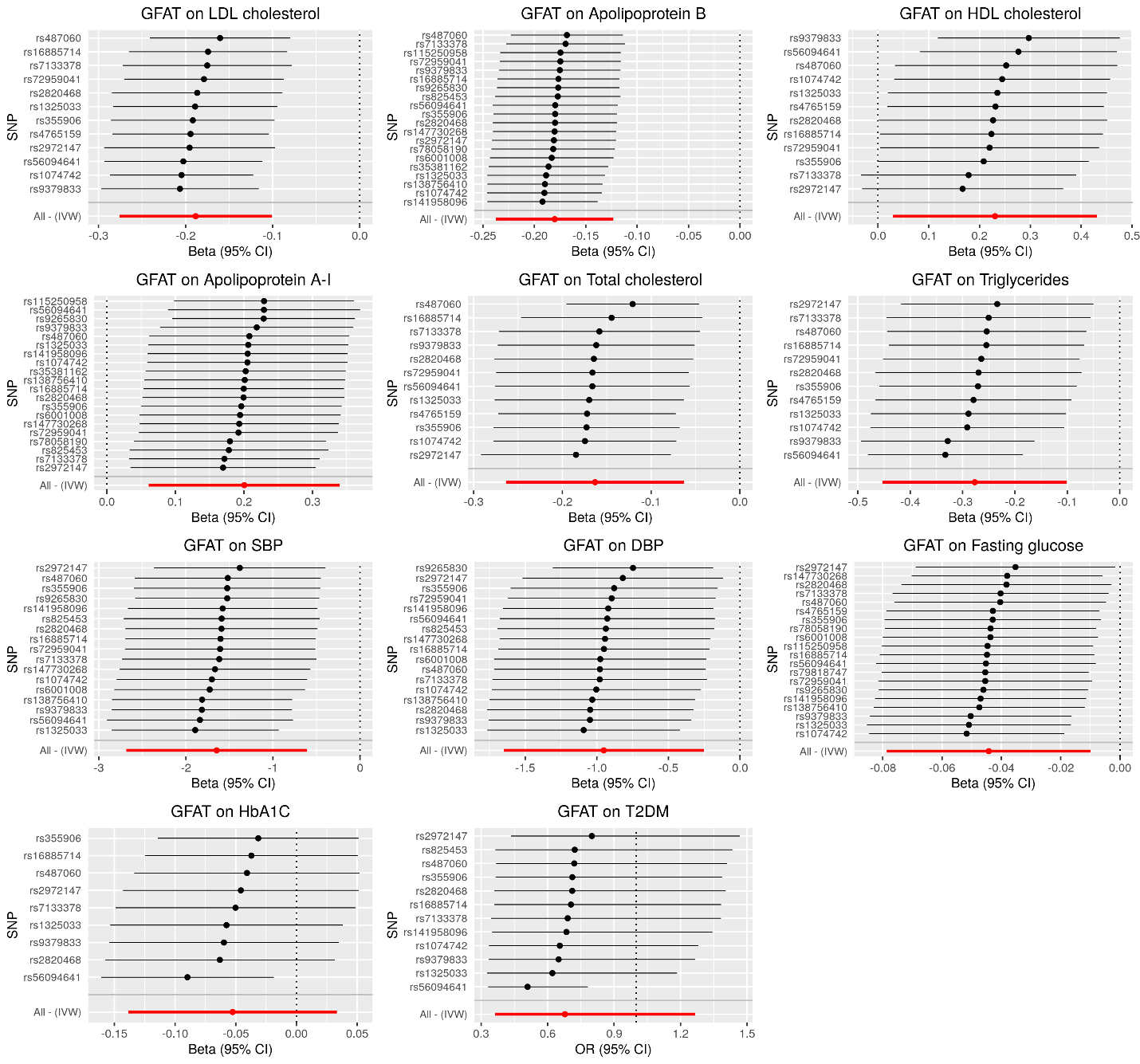


d) VAT/ASAT leave-one-out analyses.

**
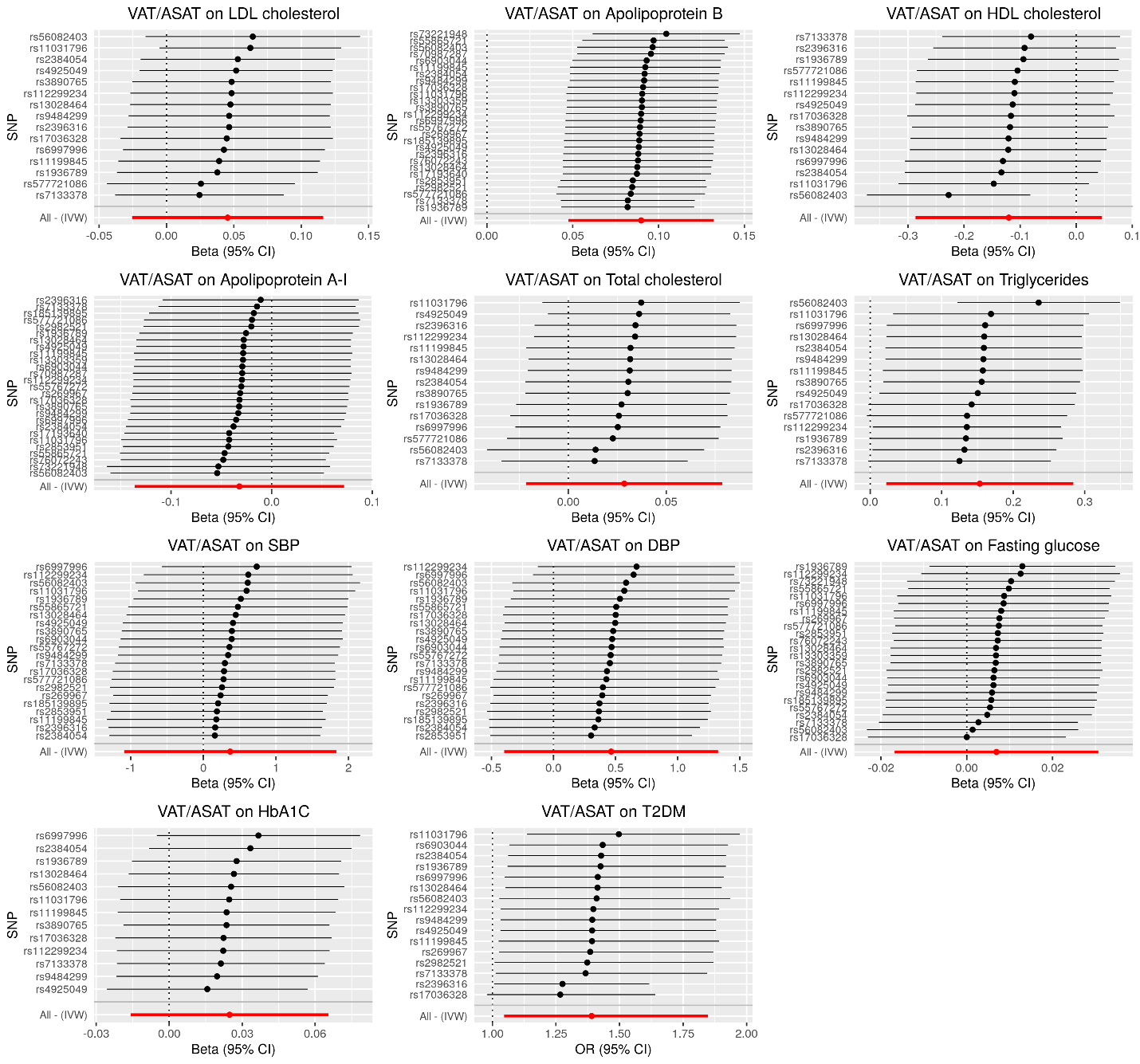
**

e) ASAT/GFAT leave-one-out analyses.
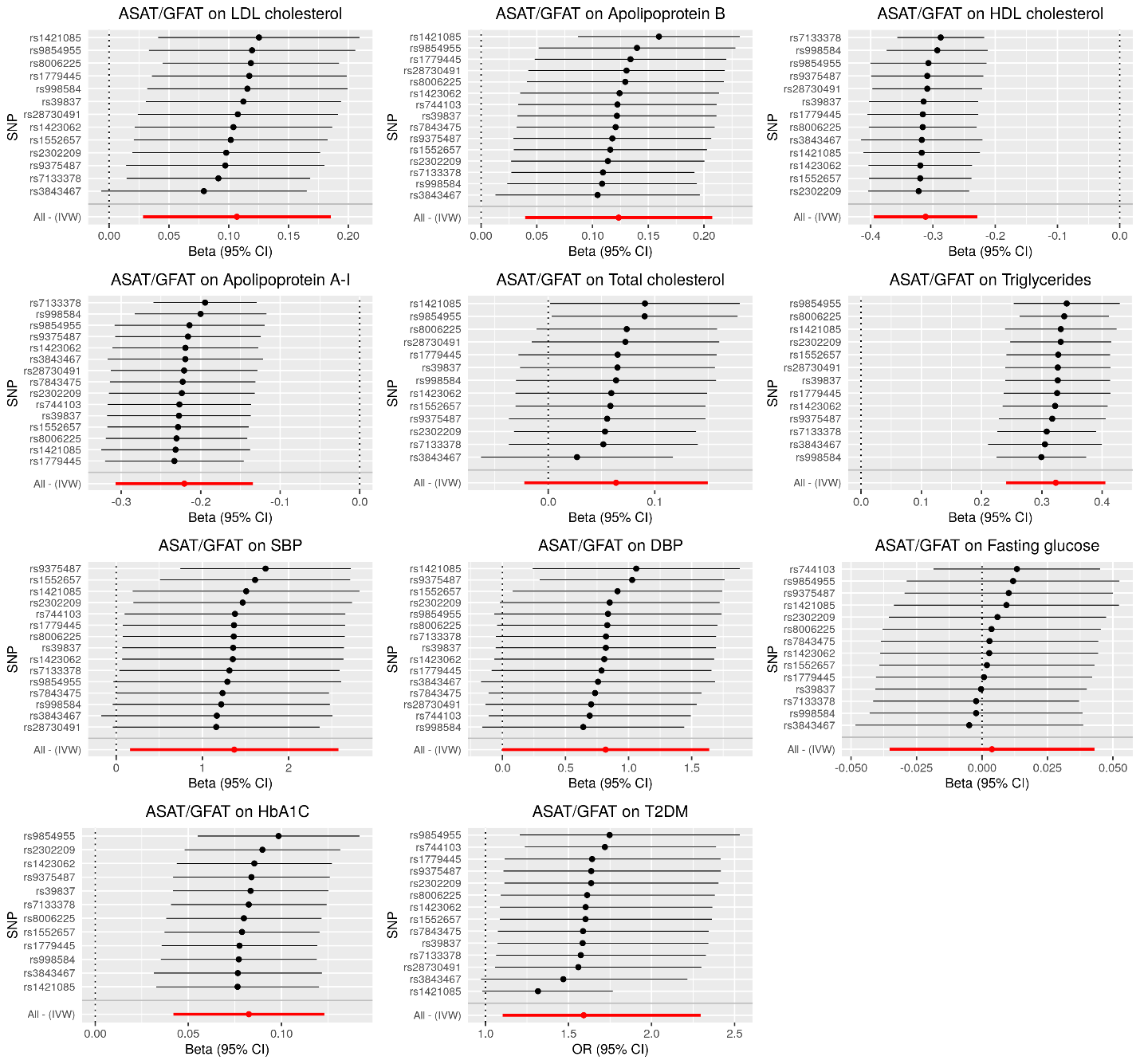


f) VAT/GFAT leave-one-out analyses.**
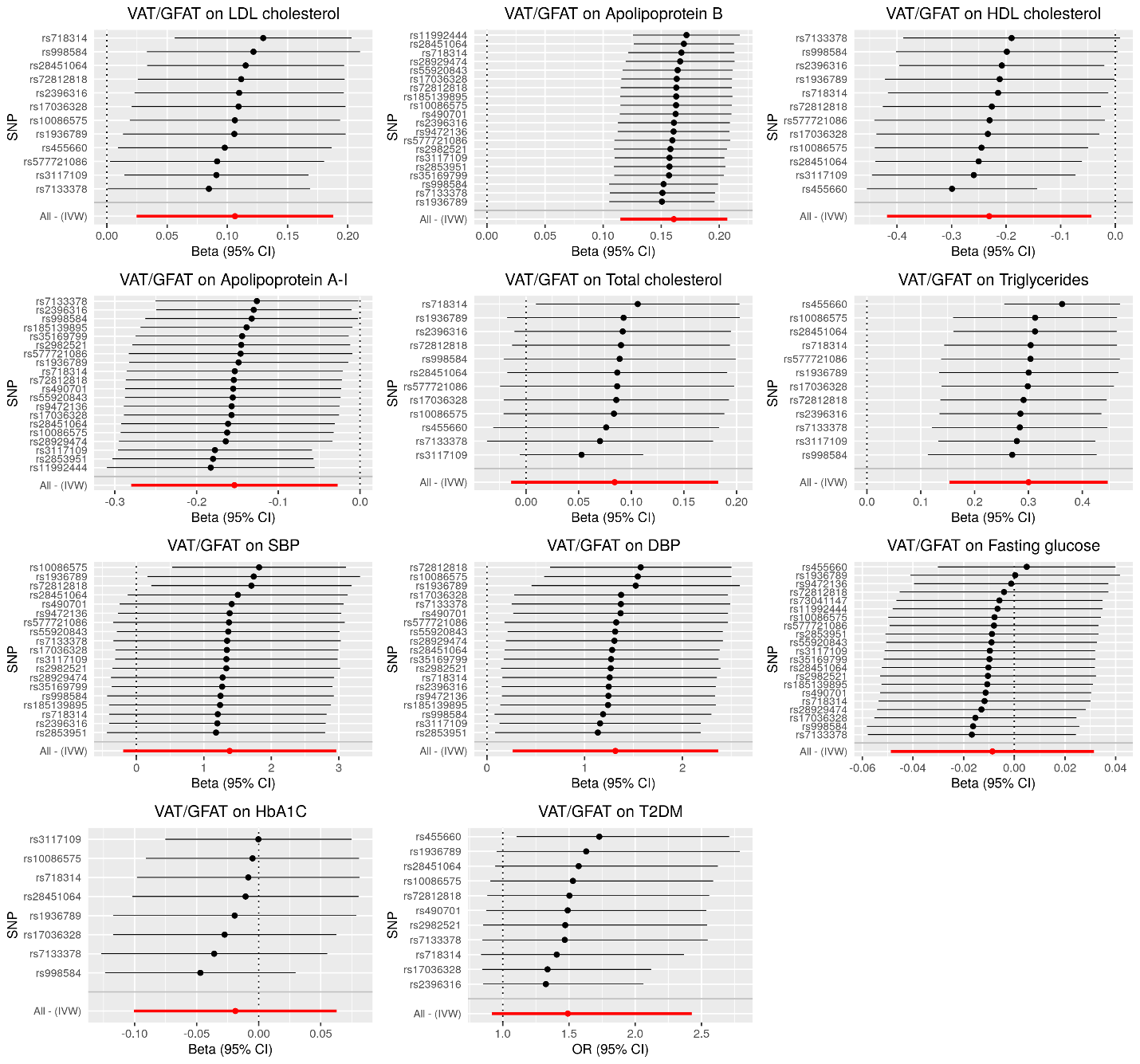
**
