# Supplementary material for: Relationships between body fat distribution and metabolic syndrome traits and outcomes: A mendelian randomization study
Source: PLoS One. 2023 Oct 26;18(10):e0293017. doi: 10.1371/journal.pone.0293017 (PMC10602264; doi:10.1371/journal.pone.0293017)

a) ASAT single SNP analyses.


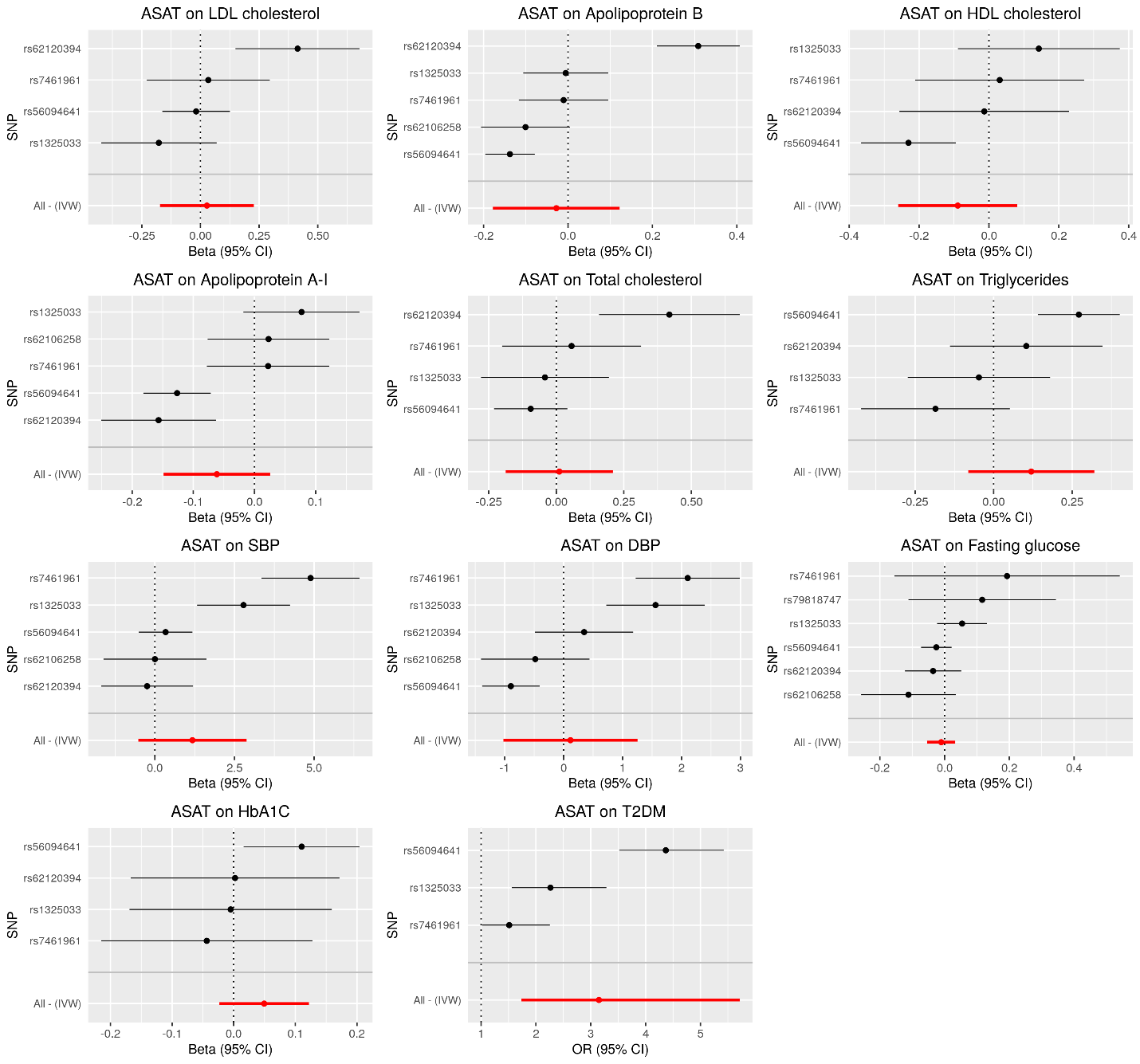


b) VAT single SNP analyses.


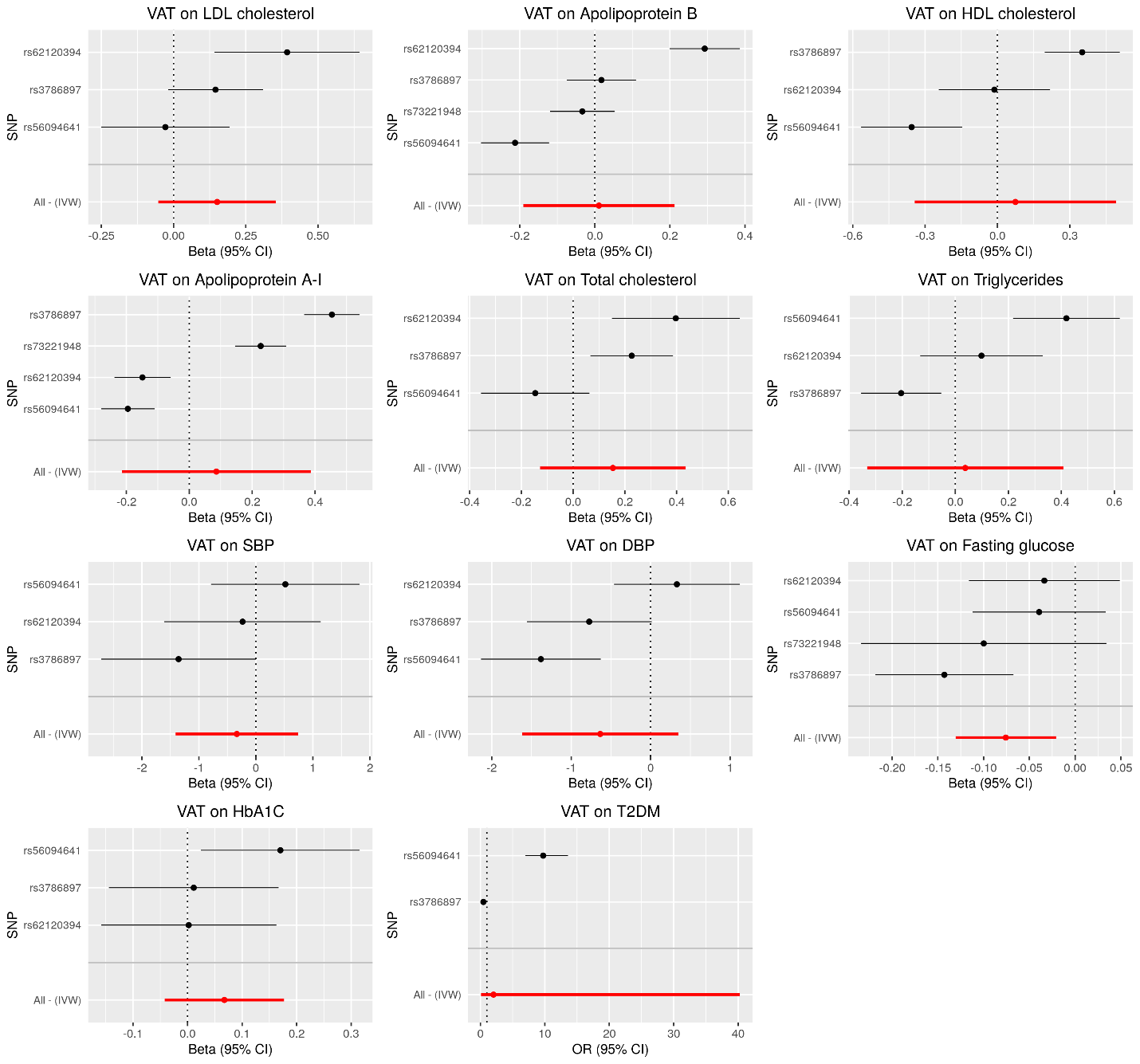


c) GFAT single SNP analyses.


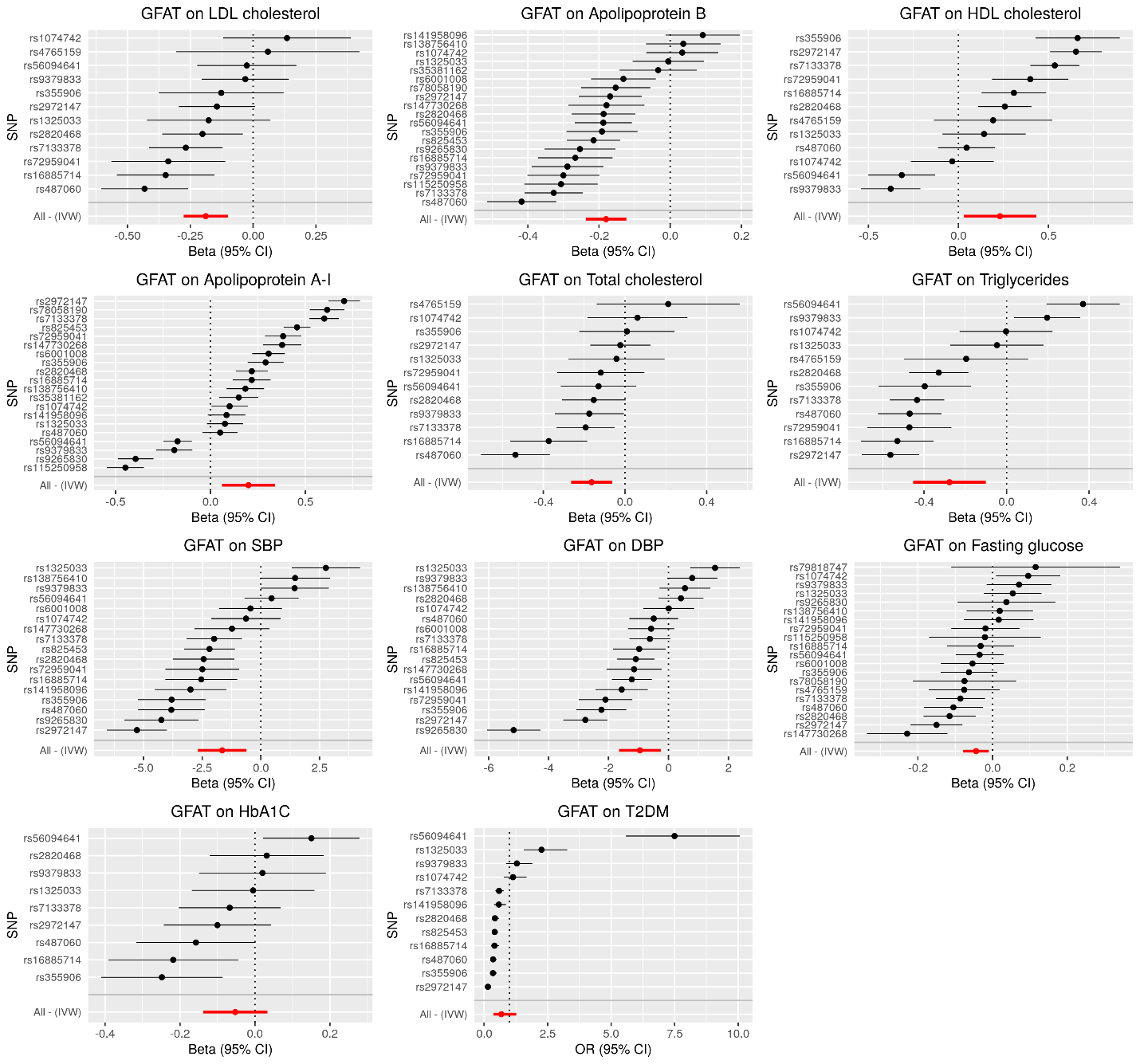


d) VAT/ASAT single SNP analyses.


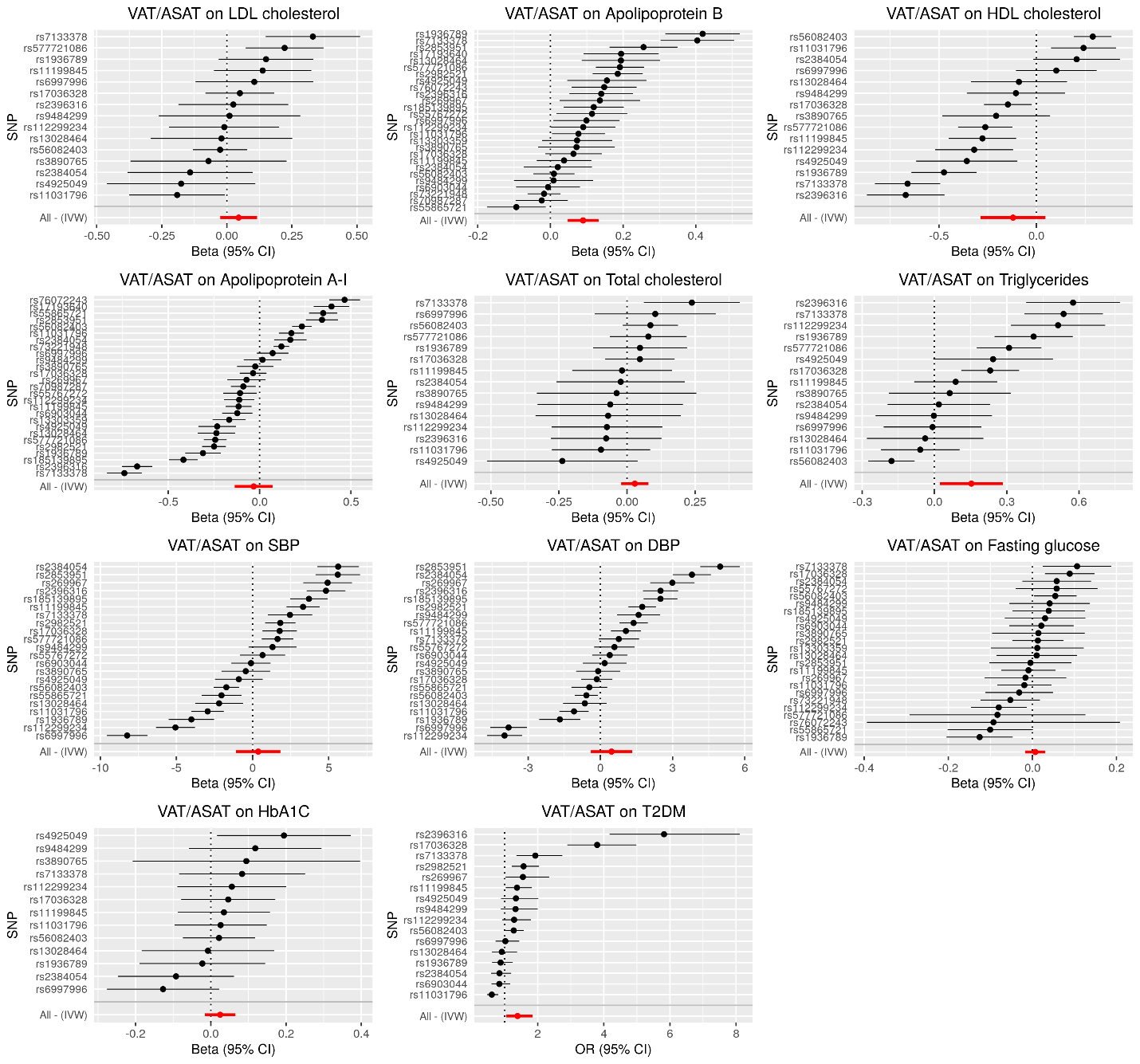


e) ASAT/GFAT single SNP analyses.


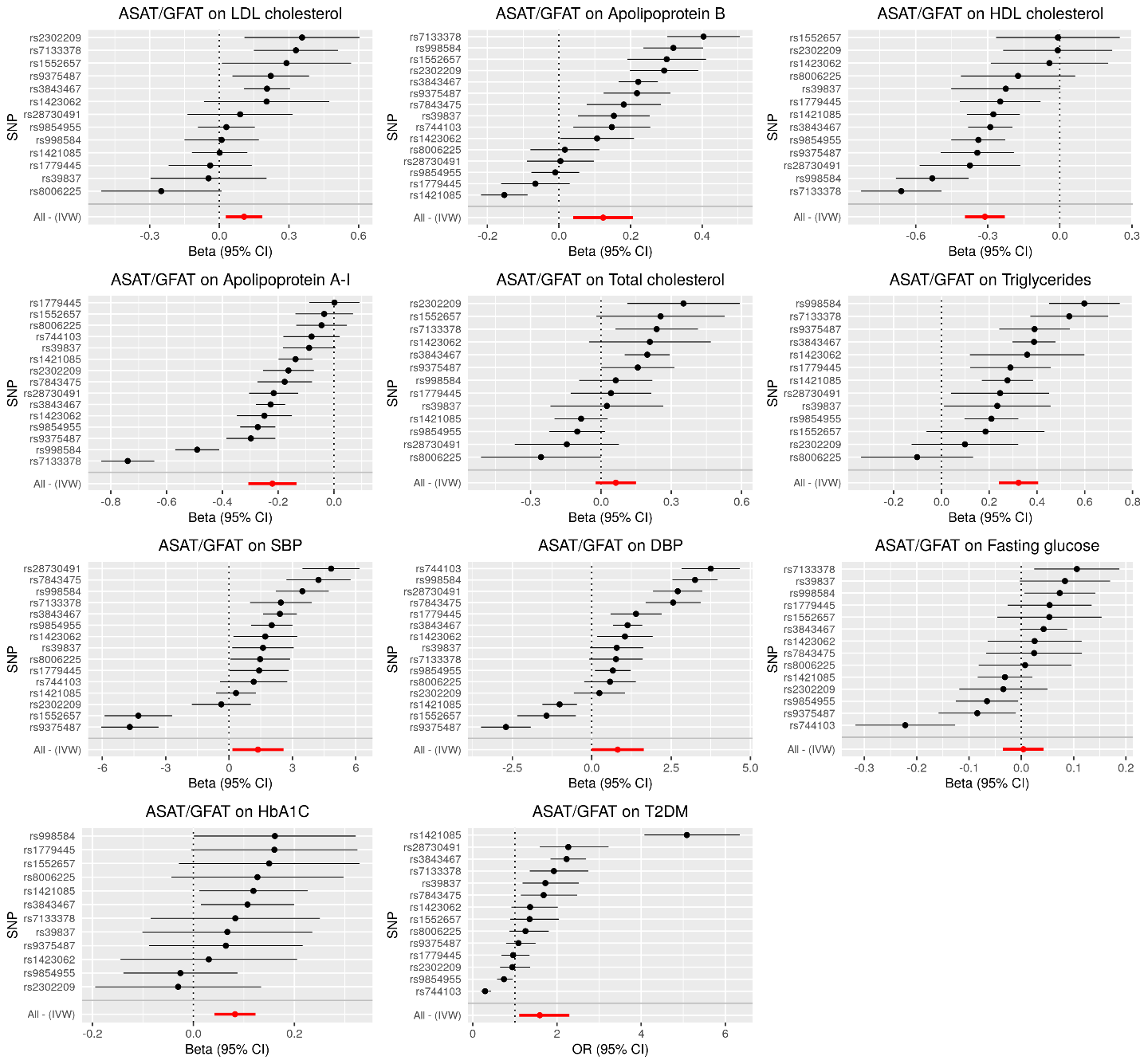


f) VAT/GFAT single SNP analyses.


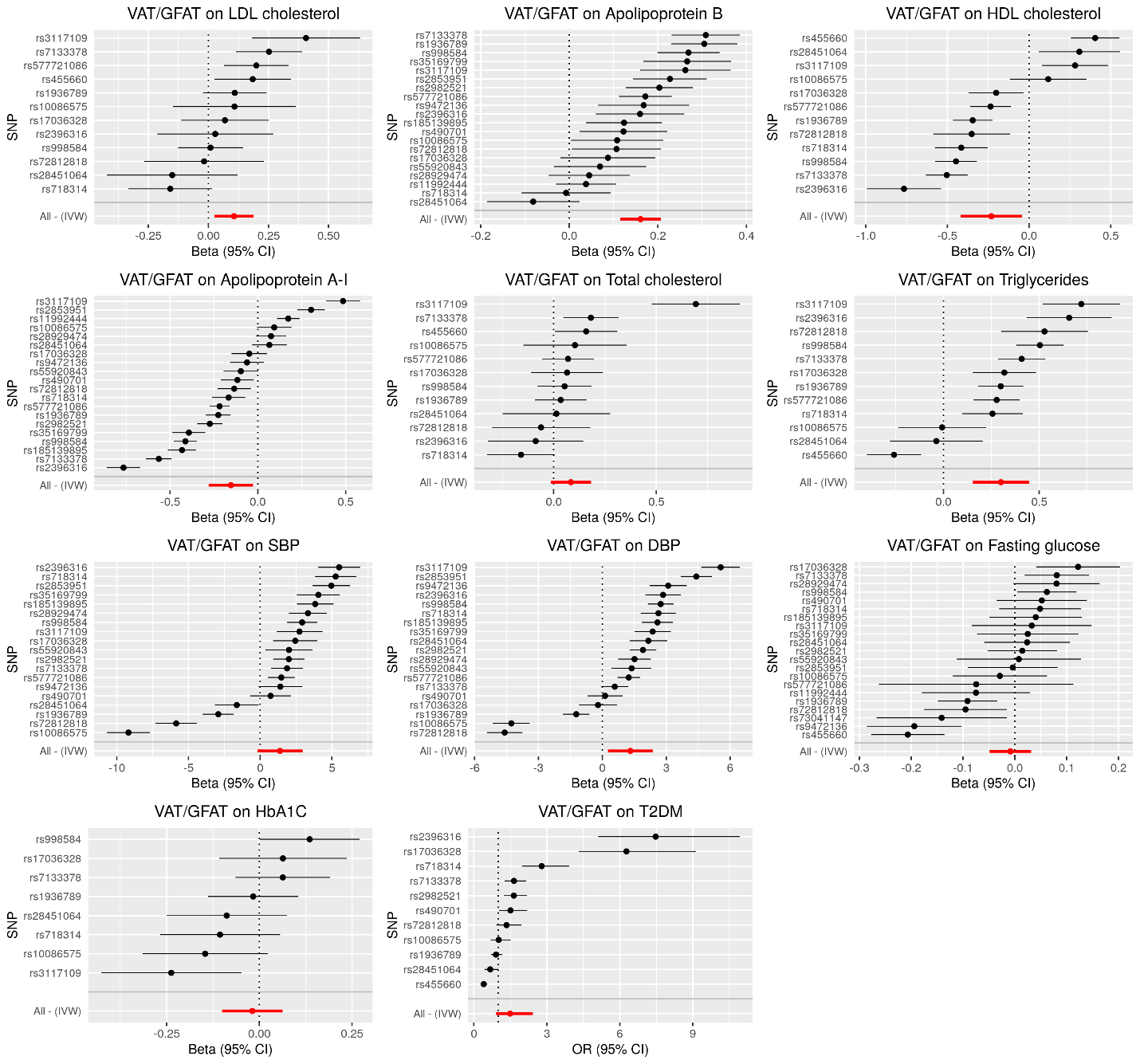

Supplement: S5 Fig — Inverse variance weighted mendelian randomization estimates were computed with single SNPs for each SNP and pair of exposures and outcomes. Estimates obtained with all SNPs included are also displayed at the bottom of each graph. ASAT, VAT, GFAT, VAT/ASAT, ASAT/GFAT, and VAT/GFAT are shown in parts a), b), c), d), e), and f) respectively. Abbreviations: ASAT (abdominal subcutaneous adipose tissue), GFAT (gluteofemoral adipose tissue), VAT (visceral adipose tissue). SBP (systolic blood pressure), DBP (diastolic blood pressure). (DOCX) [file pone.0293017.s007.docx]
