# Supplementary material for: Relationships between body fat distribution and metabolic syndrome traits and outcomes: A mendelian randomization study
Source: PLoS One. 2023 Oct 26;18(10):e0293017. doi: 10.1371/journal.pone.0293017 (PMC10602264; doi:10.1371/journal.pone.0293017)
Supplement: Attachment — Submitted filename: Response_to_reviewers.docx [file pone.0293017.s008.docx]

Dear Editor,

Thank you for the review of our manuscript. Please find our responses to each comment below along with the associated manuscript changes.

Best,

Victoria M. Gershuni, MD, MTR, MSGM

Email: victoria.gershuni@uphs.upenn.edu

Assistant Professor of Surgery

Hospital of the University of Pennsylvania

Division of Gastrointestinal Surgery

Advanced GI, Foregut, & Metabolic/Bariatric Surgery

**Reviewers' comments:**

**Reviewer #1:** Relationships between body fat distribution and metabolic syndrome traits and outcomes: a Mendelian Randomization study

The manuscript presents a comprehensive Mendelian Randomization (MR) study exploring the relationships between body fat distribution and traits or outcomes associated with metabolic syndrome. The authors have used genetic variants associated with different body fat distributions, such as visceral adipose tissue (VAT), abdominal subcutaneous adipose tissue (ASAT), and gluteofemoral adipose tissue (GFAT), identified from a Genome-Wide Association Study (GWAS) performed with the United Kingdom BioBank. The methods applied, including two-sample MR and BMI-controlled multivariable MR (MVMR), are robust, offering robust ways to explore the causal relationship between genetic determinants of fat distribution and metabolic syndrome traits. The use of summary statistics from the IEU Open GWAS Project also underscores the broad base of data used to draw conclusions.

The findings suggest a protective cardiometabolic profile associated with increased GFAT, which is maintained in analyses controlling for BMI.

The methodology section of the study provides a detailed description of the data sources, genetic instruments, and statistical approaches used in the research. The application of GWAS in identifying genetic variants associated with different fat distributions, the extraction of GWAS summary statistics for traits and outcomes related to metabolic syndrome, and the implementation of two-sample MR and multivariable MR are thoroughly explained.

Moreover, sensitivity analyses such as weighted median and MR-Egger have been included to strengthen the validity of the findings. This is commendable as these methods can provide more robust estimates under different assumptions about the presence of pleiotropy, which is a limitation in MR studies. The following could be considered to improve the manuscript.

**Response:** Thank you for your kind feedback on the analyses.

---

**Comment 1:** One area of the methodology that could have been elaborated is the specifics of the deep learning model used in predicting the ASAT, GFAT, and VAT volumes in the remaining patients. The authors mention a high r² on the hold-out test set but additional information about the type of the model, its architecture, and the performance metrics used would provide a better understanding of its validity.

**Response:** We agree that the structure and metrics of deep learning model are relevant to the underlying datasets, but since the models/analyses were not performed or altered within this study, we felt that it would be better to reference the original paper for specific details and parameters. The basic structure of the machine learning model trained by Agrawal et al. was a convolutional neural network with a DenseNet-121 architecture with pre-trained weights from ImageNet that took two-dimensional projections of the coronal and sagittal views of three-dimensional MRI images for fat and water phases, defined as the mean pixel intensity along the coronal and sagittal planes. This network was trained upon the groups of individuals with previously quantified VAT, ASAT, and GFAT (9040, 9041, and 7754 respectively) with a 80/20 split between the training and a hold-out test set. Five-fold cross-validation was performed to evaluate model performance on the training set and performance on the hold-out test set was assessed using r², achieving values of 0.991, 0.991, and 0.978 for VAT, ASAT, and GFAT respectively. We have added some of the basic details of this model to the manuscript and a reference for additional details.

**Manuscript Changes:**

**Line 97-99:** Updated the number counts for each of the patient groups.

**Line 99 -102:** Updated description of the machine learning model to “Agrawal et al. trained a convolutional neural network machine learning model on this dataset with an 80/20 training and holdout split. Five-fold cross validation was performed to assess model performance on the training set, and the model was evaluated using r^2^ on the holdout test set, achieving high r^2^ values of 0.991, 0.991, and 0.978 for VAT, ASAT, and GFAT respectively.”

**Line 104-106:** Added sentence “Full details regarding the specifics of the convolutional neural network architecture and parameters can be found within the original study.(24)”

---

**Comment 2:** It is important to note that the relationship between increased GFAT and lower levels of LDL-c, Apo-B, total cholesterol, and triglycerides, as well as higher HDL-c and Apo-A1 levels, might not necessarily mean that increasing GFAT will improve these cardiometabolic outcomes. The causality and the mechanisms behind these associations are not addressed by this study and remain to be clarified. The authors should either address this concern by either discussing it or writing it as a limitation.

**Response:** Thank you for noting the limitations of these relationships - we agree that this analysis does not imply that an increase in GFAT will lead to improvement in cardiometabolic outcomes, and there are likely many mechanisms contributing to the associations that are not able to be elucidated by this analysis. We do believe that GFAT has properties that are beneficial compared to other fat depots based on the basic science literature about these particular fat cells and the BMI controlled analyses indicates that for any given total fat volume, having a higher percentage of it in the gluteofemoral region is associated with a more protective cardiometabolic profile. However, these positive properties are in comparison to other fat depots like ASAT and VAT, and may not outweigh the negative impacts of adipose tissue more generally. We do not think a strict increase in GFAT necessarily improves any of the outcomes, although we are not able to draw any conclusions about this based on the results of this study.

We have added this discussion within limitations while referencing the mechanistic explanations from earlier in the discussion and suggested additional studies to further evaluate the observed relationships.

**Manuscript Changes:**

**Line 352-357:** Added “First, the pathophysiology behind the protective cardio-metabolic associations with GFAT are not able to be fully elucidated. These observed relationships could be from the specific metabolic properties of GFAT causing decreased fatty acid release; however, this study is not able to make a direct connection between the results and the hypothesized mechanisms.”

**Line 357-360:** Added “Furthermore, it is unclear whether some of the benefits are derived from the absence of fat deposited in more harmful adipose pockets and the hormonal and metabolic milieu associated with their deposition; it is possible that a strict increase in GFAT does not confer the same benefits.”

**Line 362-364:** Added “Further studies to assess for the effect of an isolated GFAT increase would be useful to establish a stronger causal relationship and evaluate for therapeutic potential.”

---

**Comment 3**. The data show that increased ASAT was significantly associated with increased Type 2 diabetes mellitus (T2DM) risk. However, the study could not determine whether this association is due to the adverse metabolic effects of ASAT per se or because ASAT is simply a marker for overall adiposity or other metabolic dysfunctions. This could be discussed in the discussion section or included as a limitation.

**Response:** We also agree that the mechanisms behind the association between ASAT and T2DM are not able to be determined by the analyses within the study. We think that it is likely due to a combination of increases in overall adiposity and metabolic dysfunction based on the proposed deleterious metabolic features of ASAT causing increased fatty acid turnover, although this is not a distinction able to be made based on the results from this work. We have added a line to the discussion detailing this limitation accompanying the extended discussion of limitations in elucidating GFAT mechanisms above.

**Manuscript Changes:**

**Line 365-367:** Added “Similarly, this study is not able to provide further mechanistic insight into the observed association between ASAT and T2DM, which could be from ASAT representing a general increase in adiposity or the specific metabolic features of ASAT itself.”

---

**Comment 4**: One key point is that the results show genetic associations rather than being indicative of changes over time or in response to interventions. So, while GFAT is associated with certain health outcomes, it may not be a modifiable risk factor. The authors should modify their conclusion about causation.

**Response:** Thank you for the feedback here - we agree that GFAT currently does not represent a modifiable risk factor with either lifestyle or pharmacologic interventions. Any potential therapeutic targeting of GFAT would require a better understanding of the metabolic features and mechanisms of GFAT, which this genetic study is not able to provide, and establishment of a stronger causational relationship between GFAT and a protective cardiometabolic profile. We have added lines to the limitations emphasizing this point.

**Manuscript Changes:**

**Line 360-364:** Added **“**Additionally, there is no evidence that adipose tissue can be preferentially deposited through lifestyle changes or pharmacologic interventions, so GFAT may not represent a modifiable risk factor or intervention target. Further studies to assess for the effect of an isolated GFAT increase would be useful to establish a stronger causal relationship and evaluate for therapeutic potential.”

---

**Comment 5:** The study findings indicate that absolute fat depots and fat depot ratios have differing effects on cardiometabolic health. However, the study does not explore the biological or physiological reasons behind these differences. Will be valuable to discuss the biological or physiological reasons behind these differences based on available evidence/literature.

**Response:** Thank you for commenting on this distinction, we agree that it is useful to explore the differences in absolute depots and ratios in more detail. We found that for the ASAT/GFAT and VAT/GFAT ratio analyses, the effects observed were largely consistent with what was found within the univariate analyses. Higher values of both ASAT/GFAT and VAT/GFAT, indicative of relatively lower GFAT, were associated with a more deleterious lipid profile, with the exception of total cholesterol, which was not significant.

For diabetes outcomes, both ratios had significantly increased A1c, which was not found within the univariate analyses. There were some slight differences in blood pressure as well, with SBP not being significant for VAT/GFAT and DBP not being significant for ASAT/GFAT, although the estimates are in the expected direction based on the univariate analyses.

It is difficult to discern whether this truly represents a differing effect on cardiometabolic health for ratios compared to individual depots or a result of the composite effects of each individual fat pocket. The lipid results in ASAT/GFAT and VAT/GFAT, for example, greatly resemble the univariate GFAT analysis and are likely a manifestation of the GFAT associations. Previous literature examining similar measures, such as a waist-to-hip ratio, have found similar outcomes in terms of diabetes, blood pressure, and lipids, but also do not make a clear distinction about whether this is two separate independent effects or if there is an additional interactive effect based on the ratio beyond the effects of the fat depots themselves.

Biological or physiological mechanisms for a potential additional effect are also unclear within the literature. While there are multiple proposed mechanisms for effects of each individual fat pocket, there is not a strong understanding of the interaction between different types of adipose cells that would potentially give rise to an additional effect dependent on their ratios, although the more recently proposed adipokine mechanisms could provide a potential avenue for an interactive effect. We have added sections to the limitations detailing this discussion and suggesting additional exploration given the more limited literature regarding this area.

**Manuscript Changes:**

**Line 400-407:** “The mechanisms behind the relationships seen in analysis of the fat depot ratios are also unable to identified with this analysis. The effects observed were mostly aligned with the results from each depots, suggesting they are primarily driven by the effects of the individual depots themselves. However, there is also potential for an additional interactive effect causing the ratio itself to have a differential impact, particularly given the more recently discovered adipokine signaling pathways influencing fat depot metabolism.  Prior work regarding this interaction has been limited and would be another interesting avenue of further exploration. (43,45)”

---

**Comment 6**. The study's findings are based on genetic data and might not account for environmental factors, such as diet and physical activity, which are known to affect both adiposity and cardiometabolic outcomes. This needs to be discussed or added as a potential limitation.

**Response:** We agree that there are multiple environmental and behavior risk factors that could impact the adiposity measures and the cardiometabolic outcomes, inclusive of diet, physical activity, and a number of different social determinants of health. This project was an initial exploratory analysis of these relationships; as such, we felt like examining the full extent of potential risk factors fell outside the scope of this analysis, particularly with the complexities of defining and controlling for factors such as diet and exercise. We have added this as a clear limitation and enfolded it within our updated discussion regarding the limitations of the ASAT and VAT results. One benefit of the MR methodology is that it may avoid confounding from these environmental factors; however, these environmental influences can still be a potential avenue to modify the risk factors identified through MR. As such, it would be useful to further study the connection between environmental risk factors with the fat depots and outcomes.

**Manuscript changes:**

**Line 344-345**: Added “such as environmental or behavior risk factors for obesity” as examples of a potential common confounder that may be avoided using MR

**Line 382-384:** Added “Diet, exercise, and multiple different social determinants of health including income, food availability, and transportation are all important components to obesity that are not addressed within this analysis.(53)”

**Line 396-399:** Added “Additional work examining the role that environmental factors may play in the relationships between each of these fat depots and cardiometabolic outcomes would also be beneficial and may offer further insight into potential avenues or tools to target modification in body fat distribution.”

**---**

**Comment 7:** The authors rightly mention that the GWAS utilized consists mainly of individuals aged 40-69 and self-identified as Caucasian. This limits the broad applicability of the results, considering genetic diversity and variance in obesity patterns across different ethnic and age groups. The authors should emphasize this limitation and recommend further studies involving more diverse cohorts.

**Response:** Thank you for noting these important limitations to the applicability to the results. We have added and emphasized this within the limitations section and provided a recommendation for replication in more representative cohorts.

**Manuscript changes:**

**Line 413-417:** Revised limitation discussion on general applicability to “While the sample size is large and representative of a relatively healthy UK population, the results may not be generally applicable to to all patient groups, particularly given the complex relationships between age, race/ethnicity, and obesity.(52,53) Further studies should be performed with more diverse cohorts to better assess the generalizability of these conclusions."

**---**

**Comment 8:** The authors mention that central adiposity measures (VAT and ASAT) did not show robust associations with metabolic syndrome markers in their study, which seems to contradict a significant amount of literature highlighting the deleterious effects of central adiposity. While they point out that their measures of VAT and ASAT might not have captured the full extent of abdominal adiposity, this should be investigated in more detail, and additional explanations should be considered.

**Response:** We agree that this is an important topic to explore further given the previous epidemiological studies demonstrating the deleterious associations with VAT and ASAT. We are not proposing that these results necessarily contradict what was found in previous literature and feel there may be a few different reasons that these relationships did not appear within the MR analysis. To investigate further, we computed an r-squared value for each set of the lead SNPs for each the three fat depots. The values for VAT and ASAT were on the lower end, at 0.0041 and 0.0050 respectively, indicating that there is a large amount of variation within VAT and ASAT that are not being accounted for by the lead SNPs alone. We thought this was most likely due to two main possible factors, both of which may have contributed to not detecting these metabolic associations.

First, obesity is a highly polygenic trait, and the individual fat depots are likely to be as well. As such, there is likely a large amount of genetic variation and pathways not being captured by the lead SNPs alone. The links between VAT and ASAT and the epidemiological study outcomes might be driven by these pathways and therefore not observed on the MR analysis. Additionally, there may be other upstream genetic pathways that are impacting both abdominal fat depots and the adverse cardiometabolic outcomes that are not able to be detected with the MR analysis, rather than a direct effect from the fat depots on the outcomes. Secondly, there is likely a large component of environmental influence contributing to the development of fat depots that is unable to be accounted for by a GWAS, these factors could also be contributing to the observed epidemiological relationships. Thirdly, there might be significant fat volume variation that is genetically driven but not outside the expected ranges of each depot and as such may not demonstrate the same relationships as a true pathologic increase in central adiposity.

We have added the r-squared results to the Results section and greatly expanded on the potential explanations above within the Limitations.

**Manuscript Changes:**

**Line 114:** Added “Overall r^2^ was calculated for each set of lead SNPs for each trait.”

**Line 177:** Added “The r2 values for each set of lead SNPs for ASAT, GFAT, and VAT were 0.0050, 0.0172, and 0.0041 respectively.”

**Line 374-377:** Moved “A recent large CT study of abdominal traits in 13,422 patients found strong and highly significant associations between VAT and ASAT with diabetes mellitus, hypertension, chronic kidney disease, renal failure, and renal transplant, as well as a significant increase in triglycerides, glycated hemoglobin, blood urea nitrogen, creatinine, and BMI.(52)” to Limitations section.

**Line 378-391:** Added “There are multiple reasons why we may not have observed these associations in this analysis. Firstly, the r2 values for ASAT and VAT were low, indicating that there is large amount of variation within ASAT and VAT values that are not attributable to the lead SNPs. This is likely due to the high polygenicity of obesity generally, as well as the substantial environmental influence on the development of fat depots. Unaccounted genetic pathways linking central adiposity and adverse outcomes or non-genetic environmental contributions to the epidemiologically observed relationships could both explain the lack of their detection within this analysis. Another possibility is a shared upstream genetic pathway between abdominal adiposity and adverse outcomes rather than a direct effect causing their observed associations. Additionally, there may be may be significant fat volume variation that is genetically driven but not outside expected ranges for each depot and as such may not demonstrate the same relationships as a true pathologic increase in central adiposity.”

**Line 393-396:** Revised sentence to “Future analyses using a larger GWAS of these adipose depots to capture a larger proportion of the genetic variation, or more complete measurements of central adipose tissue may better capture the expected epidemiological relationships for central adiposity.”

**Comment 9**: While the authors summarize the results of other related studies, it would add weight to their arguments if they compared and contrasted their findings more explicitly with the results of these studies.

**Response:**

Thank you for the feedback - we agree that it is useful to make a more explicit comparison with the previous studies. We identified three main bodies of results that were summarized - previous epidemiological work done on the protective effect of GFAT, previous genetics work done on body fat distribution, and epidemiological work done on abdominal adiposity, containing VAT and ASAT.

For the multiple different epidemiological studies done regarding GFAT and associated depots/measures the exact measure and outcomes varied greatly by study. As such, we directly compared and contrasted our results in line with each individual study within the Discussion.

Previous genetics studies have used anthropometric measures like waist circumference or waist-to-hip ratio rather than individual fat depots. These measures both have a component of abdominal adiposity and resulted in deleterious associations with varying metabolic outcomes, including lipids, diabetes, and blood pressure. The main difference within this study was that we examined individual fat depots and specifically highlighted the potential positive associations with GFAT with the previously studied markers of metabolic health. An additional line has been added to the discussion detailing this distinction more clearly.

For the epidemiological work done on abdominal adiposity, we have greatly expanded our discussion and provided clearer comparison and explanations as noted in the response to Comment 8. The manuscript changes for this are documented above.

**Manuscript Changes:**

**Line 275-277:** Added sentence “WHR is most closely related to the ASAT/GFAT and VAT/GFAT ratios examined in this study, which similarly demonstrated a relationship between relatively decreased GFAT and increased triglycerides and cholesterol, along with lower HDL-c.”

**Line 280-281:** Added sentence “Since GFAT is a major component of hip circumference, these epidemiological protective associations are supported by the results from this study.”

**Line 289-291:** Added sentence: “In contrast, our associations with fasting glucose and triglycerides remained when controlled for BMI, although this has many additional contributing factors beyond VAT.”

**Line 305-307:** Added sentence “Previous work has demonstrated deleterious metabolic associations with WHR and WC, both of which have a component of abdominal adiposity; this study instead highlights protective associations of GFAT on similar outcomes of lipids, blood pressure, and diabetes.”

**Reviewer #2**: The study is distinctive and novel. Mendelian randomization (MR) approach was employed by the author to investigate the causal relationship between various body fat distributions and the cardio-metabolic syndrome. Publicly available GWAS summary statistics for  different cardio-metabolic syndrome traits and  body fat distribution from IEU and  UK Biobank respectively  were used.

Large gluteal femoral adiposity was found to be cardioprotective and the association is causal, which support findings from observational studies.

**Response:** Thank you for your review of this manuscript.
